# Health-related quality of life among Chinese patients with Crohn’s disease: a cross-sectional survey using the EQ-5D-5L

**DOI:** 10.1186/s12955-022-01969-z

**Published:** 2022-04-12

**Authors:** Ziyun Gao, Pei Wang, Jie Hong, Yuqing Yan, Tianying Tong, Bin Wu, Jun Hu, Zhenhua Wang

**Affiliations:** 1 Division of Gastroenterology and Hepatology, NHC Key Laboratory of Digestive Diseases, Key Laboratory of Gastroenterology and Hepatology, Ministry of Health, State Key Laboratory for Oncogenes and Related Genes, Renji Hospital, School of Medicine, Shanghai Jiao Tong University, Shanghai Institute of Digestive Disease, 145 Middle Shandong Rd, Shanghai, 200001 China; 2 School of Public Health, Fudan University, Shanghai, 200032 China; 3 Medical Decision and Economic Group, Department of Pharmacy, Ren Ji Hospital, South Campus, School of Medicine, Shanghai Jiaotong University, Shanghai, 201112 China; 4 Department of Health Examination, Shanghai Electric Power Hospital, No. 937 West Yanan Rd, Shanghai, 200050 China

**Keywords:** Crohn’s disease, Disease activity, EQ-5D-5L, HRQoL

## Abstract

**Background:**

Crohn’s disease (CD) is a chronic disease that may have an adverse impact on health-related quality of life (HRQoL). This study aimed to describe the HRQoL of CD patients and assess correlating factors using the EQ-5D-5L in China.

**Methods:**

We recruited CD patients at Shanghai Renji Hospital from October 2018 to May 2019. The data collected included demographic and clinical information, medical expenditures, and EQ-5D-5L questionnaire responses. The chi-square test or Fisher’s exact test was applied to analyse the proportion of patients in subgroups at each level. After the selection of correlating variables by univariate analysis, multivariate regression analyses were used to explore the correlating factors of HRQoL in CD patients.

**Results:**

A total of 202 CD inpatients with a mean disease duration of 3.3 years were enrolled in the study. A total of 71.8% of patients were males, and 49.5% of patients were aged between 30 and 49 years. The average EQ-5D-5L utility score was 0.85, with a standard deviation (SD) of 0.12. Males, ileum lesions, remission status, and lower expenditure predicted higher EQ-5D-5L scores. In each EQ-5D-5L dimension, the proportion of patients differed significantly by gender, disease activity and location subgroup. In the multivariate regression models, being in an active CD state and using antibiotics had significantly adverse impacts on HRQoL (*p* < 0.05).

**Conclusions:**

CD may have a significant negative impact on HRQoL in Chinese CD patients. Being in an active phase of the disease and using antibiotics were identified as affecting HRQoL.

## Background

Crohn’s disease (CD) is one of the identified subtypes of inflammatory bowel disease (IBD), which is a chronic and incurable condition of the gastrointestinal tract [1]. CD can affect the entire intestine and regularly begins with symptoms of abdominal pain, diarrhoea and weight loss. Approximately half of patients gradually develop complications such as strictures, fistulas and abscesses [2]. During the past fifty years, the incidence of CD has increased in developed countries. Moreover, the prevalence of CD has recently been increasing in newly industrialized countries, such as China and South Korea [3, 4]. From 1982 to 2012, the number of IBD cases in China rose by approximately 2.5-fold, with a 15.7-fold increase in the number of patients with CD [5]. According to the Global Burden of Disease Study 2019, the disability-adjusted life-years (DALYs) of IBD patients in China were 232,000 (180,000–291,000) at all ages, and the age-standardized DALY rate per 100,000 was 13.1 (10.3–16.3) [6]. In general, alternation of remission and relapse places a health burden on these patients, and patients with poor health conditions have lower work capacity. The perceived stress also becomes more obvious as the disease progresses [4]. Furthermore, CD patients have financial burdens, such as health insurance costs, frequency of clinical treatment, and other expenditures [7]. Consequently, CD has negative consequences in terms of both physical and mental aspects for patients and has adverse relationships with work productivity and public health.

Health-related quality of life (HRQoL) is a multidimensional concept consisting of the physical, social and emotional aspects of health perception and functioning [8]. HRQoL can be evaluated via disease-specific and generic instruments. For CD patients, the 32-item Inflammatory Bowel Disease Questionnaire (IBDQ-32) and its short version, the Short Inflammatory Bowel Disease Questionnaire (SIBDQ) are widely used [9]. The EuroQol 5-dimensions instrument with 5-level scale (EQ-5D-5L) and Short-Form-12 Health Survey (SF-12) are the generic instruments; while the EQ-5D-5L is preference-based instrument, the SF-12 is profile-based measure of health status. The SIBDQ and SF-12 have been used to measure the HRQoL of IBD patients [10]; the EQ-5D-5L is a new version of the widely used EQ-5D-3L with improved measurement properties [11]. The increased sensitivity of the EQ-5D-5L results in smoother transitions between adjacent values and may also favour quality-adjusted life year (QALY) gains [12]. In fact, the EQ-5D-5L has better validity than the EQ-5D-3L with regard to feasibility, the ceiling effect, and discriminatory power for CD [13].

Patients with severe IBD were found to have impaired HRQoL in an Australian population [14]. Among patients with IBD in the US and five European countries, active disease was shown to be related to significant impairments in HRQoL, as well as work and leisure activities [15]. In Asian countries, a higher population density is related to a higher prevalence of CD. In China, especially in coastal areas, the incidence of CD is positively associated with the gross domestic product [16]. In China, most patients seek health care in tertiary-care public hospitals in large cities, which predominate in the country. These hospitals are well equipped to address chronic and complex diseases such as CD. However, it is still not convenient for all CD patients to obtain specialist treatment [17]. However, relevant analyses of CD patients using the EQ-5D-5L are rare, especially for Asian countries such as China.

The objective of our study was to measure HRQoL in Chinese CD patients using the EQ-5D-5L and evaluate how demographic and clinical features affect HRQoL. We hypothesized that female sex, older age, and more severe disease would have a negative impact on HRQoL.

## Methods

### Study design and patients

**Fig. 1:**
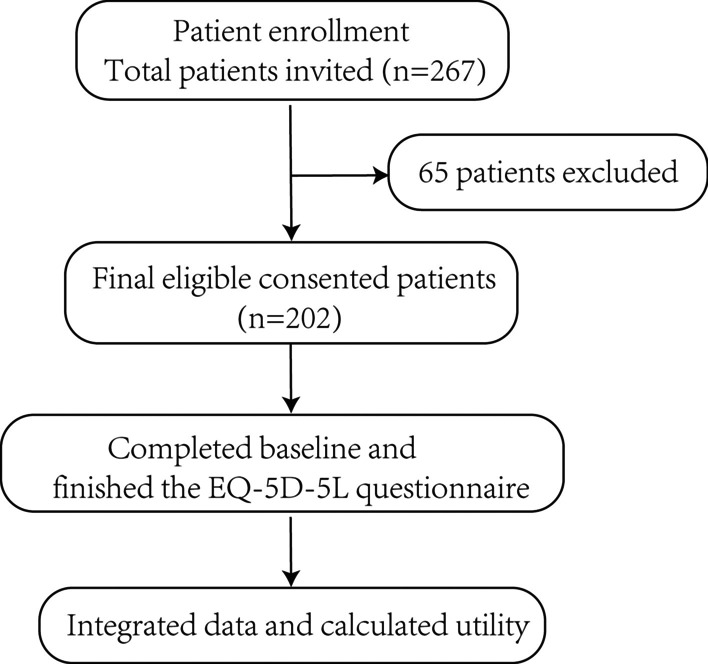
Flow chart of the study

This cross-sectional study was conducted at Shanghai Renji Hospital, a tertiary-care public hospital staffed with IBD specialists, from October 2018 to May 2019 (Fig. 1). The sample size was calculated based on the formula Z_1__–α/2_^2^ p(1–p)/d^2^ [18]. Z_1–α/2_ is the standard normal variate which is 1.96 with a 95% level of confidence, and d indicates 5% of a margin of error. According to a study that reported the global burden of IBD, China would have over 1.5 million cases of IBD, and the prevalence would plateau at 0.1% [19]. Thus, the expected sample size was 1.5. Considering the relatively rare incidence of CD in China, we recruited more CD patients than the calculated sample size. The inclusion criteria were formulated according to the “ACG Clinical Guideline: Management of Crohn’s Disease in Adults” and included the following: (1) clinical symptoms such as chronic diarrhoea, abdominal pain, weight loss and fatigue; (2) ileocolonoscopy with biopsy showing mucosal changes, such as mucosal nodularity, oedema, ulcerations, friability, and stenosis; (3) computed tomography enterography (CTE) and magnetic resonance enterography (MRE) indicating complications, such as enteric stricture and fistulas; and (4) laboratory tests, such as C-reactive protein (CRP) and erythrocyte sedimentation rate (ESR), demonstrating inflammatory activity [20]. Another inclusion criterion was that the patients had the ability to understand the EQ-5D-5L questionnaire and were willing to complete it. The exclusion criteria were any other diseases with similar symptoms or affecting HRQoL, such as ulcerative colitis, Behcet’s disease, intestinal lymphoma, intestinal tuberculosis, ischaemic colitis or intestinal infectious diseases.

### Study procedure

Before the survey, verbal consent was obtained from all patients, and all were fully informed of the purpose, process and rights regarding data collection. Consenting patients were asked to self-complete the EQ-5D-5L questionnaire and disease-related questions, including those on height, weight, the Crohn’s Disease Active Index (CDAI), disease duration and location, and medications. Sociodemographic characteristics, including gender and age, were gathered from the medical record system. The CRP and ESR results were collected from laboratory test reports.

All procedures were performed in accordance with the ethical standards of the Research Ethics Committee of Shanghai Jiao Tong University School of Medicine, Renji Hospital, and our study was approved by the Ethics Committee of [2015] 097.

### The EQ-5D-5L

The EQ-5D-5L consists of five dimensions and five levels forming a matrix of 3125 possible health states. The five dimensions include mobility (MO), usual activities (UA), self-care (SC), pain or discomfort (PD), and anxiety or depression (AD). The five levels rating HRQoL in each dimension are (1) no problems, (2) slight problems, (3) moderate problems, (4) severe problems and (5) extreme problems [21]. All health states can be converted into a single utility index score, which can be obtained by subtracting the weight in each dimension from 1(fully healthy). A negative value means that the health state was worse than death (score = 0).

In this study, the Chinese EQ-5D-5L value set established in 2017 was adopted to calculate the utility score [22].

### Explanatory variables

Several demographic and clinical characteristics have been identified as being related to the HRQoL of CD patients, including age, gender, education level, disease duration, location, and clinical activity [23]. Three kinds of variables, including sociodemographic data (i.e., age and gender), clinical information (BMI, location and duration of CD, and disease activity indices), and expenditures, were adopted to assess associations with HRQoL. Disease activity indices include the CDAI, ESR and CRP; disease locations include ileal lesions (L1), colonic lesions (L2), ileocolonic lesions (L3), and upper-isolated gastrointestinal lesions (L4) according to the Vienna classification and the Montreal classification for Crohn’s disease [1]. The standard activity level was the CDAI, ranging from 0 to 600. A score lower than 150 indicated disease remission, a score from 150 to 450 was considered to indicate an active state, and a score more than 450 was considered to indicate extremely severe disease. Current medications comprised 5-aminosalicylates, corticosteroids, immunomodulators, biologics and antibiotics. Data on unit expenditures, including medical services, diagnostic costs and medications, were collected during hospitalization. Expenditures were divided into four levels based on the interquartile range, including less than ¥ 8,000, ¥ 8,000 to 16,000, ¥ 16,000 to 24,000 and more than ¥ 24,000.

### Data analysis

Descriptive statistics were used to analyse sociodemographic and clinical characteristics as well as medical expenditures. Categorical variables are summarized as frequencies and percentages. Continuous variables are presented as the mean and standard deviation (SD). The EQ-5D-5L utility scores among subgroups were assessed and compared using univariate Tobit models. The chi-square test or Fisher’s exact test was applied to compare the proportion of patients in subgroups at each level.

To evaluate factors correlating with HRQoL in CD patients, univariate regression models were separately adopted in three kinds of multivariate regression models. Significant variables with *p* < 0.20 were further included in multivariable regression models. The first was the ordinal least squares (OLS), which is a classical regression model used to examine health utility, as in a previous study [24]. In our study, the range of the EQ-5D-5L utility score was from 0.493 to 1, and there were 21 patients who reported that they were in full health. The Tobit regression model was then adopted to analysis. In addition, patients with an above-average utility score (0.85) were regarded as having a relatively better HRQoL, and logistic regression was applied to predict the probability of patients reporting better health. In the regression model, 0 and 1 indicated utility less than 0.85 or greater than/equal to 0.85, respectively. After the multivariable analysis, type I errors were adjusted, and the variance inflation factor (VIF) was used to assess multicollinearity. In addition, the Akaike information criterion (AIC) was employed to estimate the overall fit of the models.

All statistical analyses were performed using R (Version 4.0.4). The statistical significance in this study was set at the 5% level.

## Results

### Clinical characteristics of patients with Crohn’s disease

Among the 267 patients recruited, 202 eligible CD patients were identified and participated in the survey. There were 145 (71.8%) males in our study, and the mean age of all patients was 31 years. The most common location was L3 (ileocolonic lesions, 43.1%), followed by L1 (ileal lesions, 42.6%). The mean CDAI was 159.4 (SD = 47.40). A total of 38.1% of the patients were in remission; the other 61.9% were in an active disease state. The mean disease duration was 3.30 years (SD = 3.41). Many patients used antibiotics (29.5%); corticosteroids were the least used (13.6%). Mean expenditures amounted to RMB 17,380.00 (SD = 11,520.00) per patient on average (Table 1).

**Table 1:** Sociodemographic characteristics of the respondents

Variables	N	%	Mean	SD
Overall	202			
*Gender*				
Male	145	71.8		
Female	57	28.2		
*Age group, years old*			31.00	9.69
< 30	95	47.0		
30–49	100	49.5		
≧ 50	7	3.5		
*CD location*				
L1	86	42.6		
L2	28	13.9		
L3	87	43.1		
L4	1	0.5		
*CDAI*			159.40	47.40
≦ 150	77	38.1		
150–450	125	61.9		
*Duration of CD, years*			3.30	3.41
BMI, kg/m^2^			20.10	3.20
ESR, mm/h			22.00	20.00
CRP, mg/L			19.60	24.60
*Medications*				
5-Aminosalicylates	45	17.4		
Corticosteroids	35	13.6		
Immunomodulators	45	17.4		
Biologics	57	22.1		
Antibiotics	76	29.5		
*Expenditure, RMB*			17,380.00	11,520.00
< 8000	49	24.3		
8000–16,000	52	25.7		
16,000–24,000	49	24.3		
> 24,000	52	25.7		

*SD* standard deviation, *CD* Crohn’s Disease, *CDAI* Crohn’s Disease Active Index, *BMI* Body Mass Index, *ESR* erythrocyte sedimentation rate, *CRP* C-reactive protein

### EQ-5D-5L distribution

**Fig. 2:**
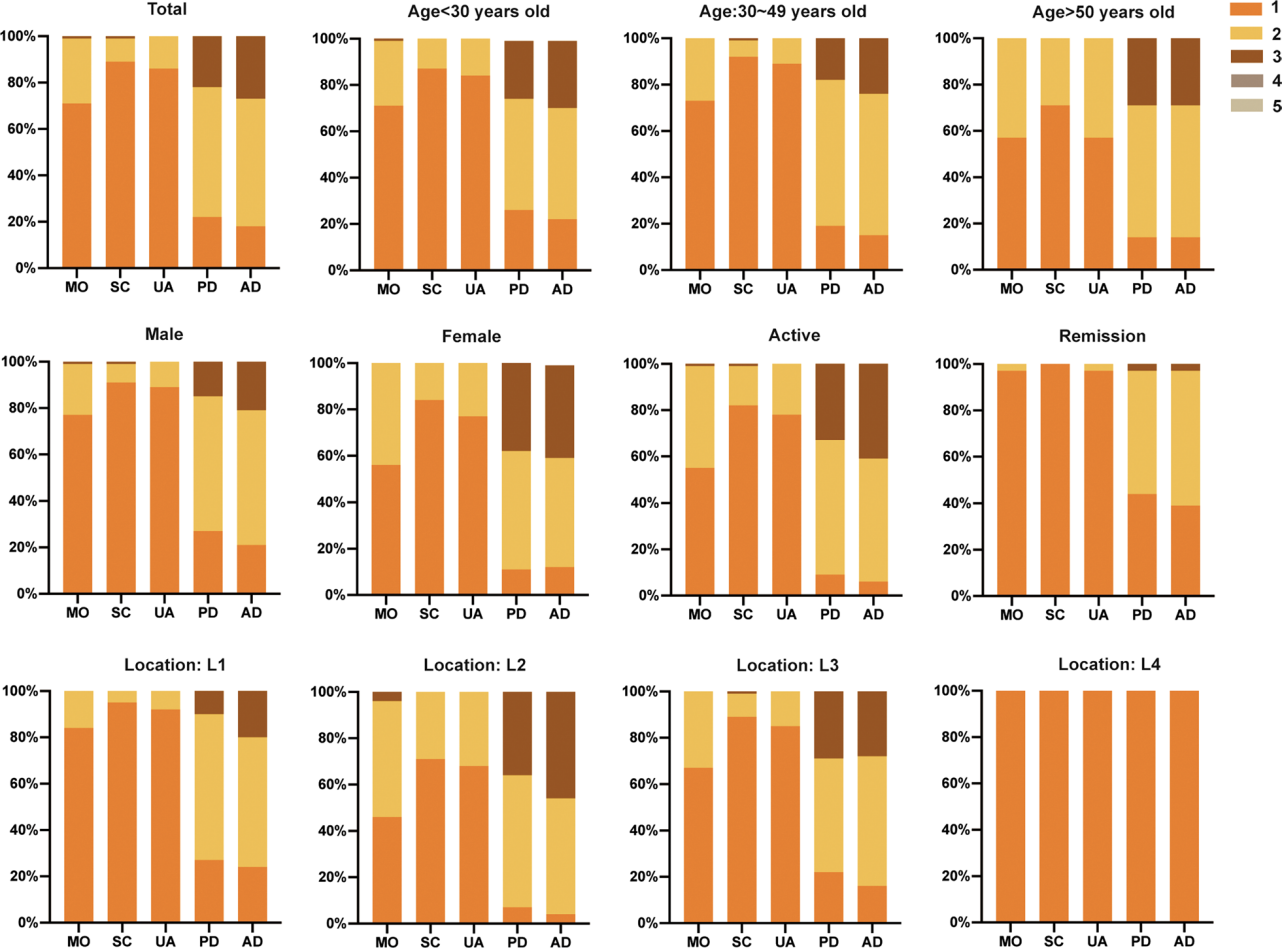
Proportion of patient responses by extent of problems in each EQ-5D-5L dimension by social-demographic groups

The score distribution and percentage in each level of the subgroups are shown in Fig. 2. In total, CD patients reported worse health in the pain/discomfort and anxiety/depression dimensions than in the other three EQ-5D dimensions. In subgroups, the proportion of patients reporting health problems in the pain/discomfort and anxiety/depression dimensions increased gradually with age. In addition, females generally had more health problems than males. There were significant differences between males and females in mobility (level = 1, *p* < 0.01; level = 2, *p* < 0.01), pain or discomfort (level = 1, (*p* = 0.02; level = 3, *p* < 0.01) and anxiety or depression (level = 3, *p* = 0.01). Compared with those in remission, patients in an active disease state reported a poorer health status in all dimensions. Regarding location, CD patients who had colonic lesions had the most health problems. Except for mobility (level = 3), self-care (level = 3), pain or discomfort (level = 2) and anxiety or depression (level = 2), there was a significant difference between the CDAI and location groups (*p* < 0.05).

### EQ-5D-5L utility

The average EQ-5D-5L utility score was 0.85 (SD = 0.12). In the subgroup analysis, males (mean: 0.87) had higher scores than females (mean: 0.80), and the utility score for those between 30 and 49 years old (mean: 0.85) was higher than that of other age groups. Ileal lesions (mean: 0.88) had the highest score in the location group, except for upper-isolated gastrointestinal lesions, which were reported by only one patient. Being in the remission state had a higher utility score (mean: 0.93) than being in the active phrase (mean: 0.80). Patients who used corticosteroids had the lowest utility score (mean: 0.81), and those using biologics had the highest score (mean: 0.92). With regard to health care costs, spending over RMB 24,000 had the lowest utility score (mean: 0.77). Overall, there were statistically significant differences between subgroups, including by gender (*p* < 0.05), the CDAI (*p* < 0.01), medications (*p* < 0.05) and expenditure (*p* < 0.05). The descriptive statistics of the utility score are shown in Table 2.

**Table 2:** Descriptive statistics of the EQ-5D-5L utility

Variables	Mean	SD	Median	Q1	Q3	IQR	Q3-Mean	P^a^
Overall	0.85	0.12	0.89	0.78	0.94	0.16	0.09	
*Gender*								** < 0.05**
Male	0.87	0.11	0.89	0.82	0.95	0.13	0.08	
Female	0.80	0.12	0.82	0.74	0.89	0.15	0.09	
*Age group*								> 0.05
< 30	0.85	0.13	0.89	0.75	0.95	0.21	0.11	
30–49	0.85	0.10	0.89	0.81	0.89	0.08	0.04	
≧ 50	0.80	0.15	0.89	0.68	0.92	0.23	0.11	
*CD location*								> 0.05
L1	0.88	0.09	0.89	0.82	0.95	0.13	0.07	
L2	0.77	0.13	0.82	0.72	0.84	0.13	0.07	
L3	0.84	0.12	0.89	0.75	0.94	0.20	0.11	
L4	1.00	0.00	1.00	1.00	1.00	0.00	0.00	
*CDAI*								** < 0.01**
≦ 150	0.93	0.05	0.94	0.89	1.00	0.11	0.07	
150–450	0.80	0.12	0.82	0.74	0.89	0.15	0.10	
*Medications*								** < 0.05**
5-Aminosalicylates	0.83	0.13	0.84	0.74	0.89	0.15	0.07	
Corticosteroids	0.81	0.13	0.83	0.75	0.89	0.15	0.08	
Immunomodulators	0.84	0.12	0.89	0.78	0.89	0.11	0.05	
Biologics	0.92	0.07	0.89	0.89	0.95	0.06	0.04	
Antibiotics	0.82	0.13	0.82	0.68	0.89	0.22	0.07	
*Expenditure, RMB*								** < 0.05**
< 8000	0.92	0.08	0.94	0.89	0.95	0.06	0.03	
8000–16,000	0.78	0.09	0.89	0.83	0.94	0.12	0.16	
16,000–24,000	0.84	0.11	0.83	0.76	0.89	0.14	0.05	
> 24,000	0.77	0.13	0.80	0.65	0.89	0.25	0.13	

*SD* standard deviation, *IQR* interquartile range, *CD* Crohn’s Disease, *CDAI* Crohn’s Disease Active Index

P^a^: P value was calculated from mean

### Association between the EQ-5D-5L utility score and patient characteristics

The EQ-5D-5L utility score was a continuous outcome. According to univariate models, gender, CDAI, BMI, ESR, CRP, medications and expenditure were analysed in three regression models (i.e., OLS, Tobit and logistic regression). In our study, a VIF < 10 indicated no collinearity issues in the multicollinearity test. In OLS regression, the utility score of being in an active disease state and the usage of antibiotics decreased by 0.089 (*p* = 0.000) and 0.053 (*p* = 0.011), respectively. In the Tobit model, the association was similar to the above models; only the CDAI and usage of antibiotics had a significantly adverse impact on HRQoL. In logistic regression, being in an active disease state (OR = 0.036, *p* = 0.000) and using antibiotics (OR = 0.198, *p* = 0.032) were negatively associated with HRQoL. Other variables showed no statistical significance with the EQ-5D-5L utility score (*p* > 0.05). The AIC values of these models were − 357.32, − 254.65 and 147.37 when estimating the overall fit of the models. The regression analyses are shown in Table 3.

**Table 3:** Ordinal least squares (OLS), Tobit regression and Logistic regression for EQ-5D-5L utility

Variables	Univariate			OLS			Tobit			Logistic regression		
	β	SE	P	β	SE	P	β	SE	P	OR	SE	P
*Gender*												
Female	− 0.067	0.018	**0.000**	− 0.027	0.015	0.258	− 0.029	0.016	0.219	1.109	0.536	0.923
*Age group*												
30–49	0.008	0.017	0.620									
≧ 50	− 0.040	0.046	0.379									
*CD location*												
L2	− 0.110	0.024	0.000									
L3	− 0.045	0.017	0.008									
L4	0.118	0.112	0.293									
*CDAI*												
150–450	− 0.135	0.014	**0.000**	− 0.089	0.015	**0.000**	− 0.104	0.016	**0.000**	0.036	0.756	**0.000**
Duration of CD	0.003	0.002	0.298									
BMI	0.012	0.002	**0.000**	0.002	0.002	0.662	0.003	0.002	0.533	1.165	0.080	0.136
ESR	− 0.002	0.000	**0.000**	0.000	0.000	0.810	0.000	0.001	0.837	0.957	0.021	0.119
CRP	− 0.002	0.000	**0.000**	0.000	0.000	0.662	0.000	0.000	0.718	1.020	0.014	0.287
*Medications*												
5-Aminosalicylates	− 0.036	0.020	**0.070**	− 0.009	0.016	0.766	− 0.006	0.017	0.869	1.055	0.574	0.925
Corticosteroids	− 0.044	0.021	**0.040**	− 0.016	0.018	0.662	− 0.015	0.019	0.658	0.787	0.616	0.852
Immunomodulators	− 0.020	0.020	0.323									
Biologics	0.089	0.017	**0.000**	0.011	0.018	0.766	0.020	0.020	0.633	2.906	0.759	0.287
Antibiotics	− 0.113	0.015	**0.000**	− 0.053	0.017	**0.011**	− 0.056	0.018	**0.011**	0.198	0.581	**0.032**
*Expenditure*												
8000–16,000	− 0.045	0.021	**0.031**	0.004	0.018	0.848	− 0.001	0.020	0.977	1.662	0.723	0.056
16,000–24,000	− 0.078	0.021	**0.000**	− 0.009	0.020	0.782	− 0.009	0.022	0.908	0.598	0.714	0.707
> 24,000	− 0.149	0.021	**0.000**	− 0.055	0.022	0.055	− 0.058	0.023	0.056	0.753	0.765	0.852
AIC				− 357.32			− 254.65			147.37		

Reference level: male, age < 30 years old, L1, CDAI ≤ 150, 5-Aminosalicylates (No), Corticosteroids (No), Immunomodulators (No), Biologics (No), Antibiotics (No), expenditure < RMB 8,000

*OLS* ordinal least squares, *AIC* Akaike information criterion, *CDAI* Crohn’s Disease Active Index

## Discussion

To our knowledge, this is the first study using the EQ-5D-5L to evaluate the HRQoL of people with CD in China. This cross-sectional study indicates that CD could impair patients’ HRQoL measured by the EQ-5D-5L. All three regression analyses showed that being in an active disease state and using antibiotics were negatively associated with HRQoL. Consequently, patient-oriented management merits widespread attention. CD patients need better health care management, which should be included in routine clinical practice to inform physicians about patients’ perception of their health.

In our sample, the mean EQ-5D-5L utility score was 0.85, which is lower than the EQ-5D-5L norms in China (mean score: 0.96) [25]. Although more patients in an active disease state were recruited, no one reported having “severe or extreme problems”, indicating that they did not have many problems in the EQ-5D dimensions. The mean score was 0.93 for remission and 0.80 for the active state in our study, and HRQoL in the former was better than that in the latter in all EQ-5D-5L dimensions (Fig. 2). In the regression model, we found that being in an active state impaired the HRQoL of CD patients. Consequently, CD activity is quite crucial to the quality of life. In line with previous research, HRQoL in individuals both in the active disease and remission periods was significantly worse than that in healthy people, and the health state in those with active disease was more severe [26–28]. The CDAI is associated with more threatening experiences, less satisfaction and reduced HRQoL [29]. In Singapore and Brazil, clinical characteristics such as CD activity are associated with a substantial impact on HRQoL, work productivity impairment and an increased number of IBD surgeries and hospitalizations [30, 31].

Disease activity is considerably connected to psychological problems in CD patients [32]. When a disease relapses, symptoms of anxiety and depression are more likely to appear [33]. Our findings also revealed that more patients reported “moderate problems” in the anxiety/depression dimension (Fig. 2). Consistent with our study, scholars in Brazil also believed that anxiety played a predominant role in patients’ mental burden, followed by anxiety concomitantly with depression, whereas depression alone had the least impact [33]. In China, Luo et al. reported that 24.7% of patients had anxiety and that 17.4% were affected by depression [34]. Owing to the uncertain aetiology and recurrent nature of CD, overcoming mental stress is quite difficult for the majority of patients. Previous research has shown that a higher level of perceived stress is a strong predictor of lower HRQoL, which may cause low adherence to provider recommendations [35]. Thus, illness perceptions and mental stress mediated disease severity and HRQoL. These factors should be incorporated into coping strategies [36, 37]. On the other hand, awareness of such experiences and the ability to live with CD may be helpful for eliminating stress [38]. However, only a few studies have supported the popularization and effectiveness of psychopharmacological treatments. It seems reasonable to implement such therapy, but further exploration is needed [39]. In our study, females experienced worse anxiety or depression, which was significantly different from males, in line with a former study showing that females had a higher recurrence rate and were more likely to worry about being a burden on their families [40]. Consequently, psychological disorders are worthy of our attention.

In our study, higher expenditures strongly reduced EQ-5D-5L utility scores. Direct medical expenditures consist of medical services, diagnostic costs, and medications, with medications having the greatest cost proportion. Individuals with CD may use multiple drug regimens, including 5-aminosalicylates, corticosteroids, immunomodulators, biologics, and antibiotics, for synergistic management of their condition. In our study, the usage of antibiotics had an adverse impact on the HRQoL of CD patients, while a study found the corticosteroids led to worse effects[30]. This might be due to differences in drug selection and the population. Moreover, taking multiple medications suggests more severe disease symptoms or complications [41]. The financial and mental burden may further exaggerate dissatisfaction and thus lead to a negative effect on HRQoL.

In developing countries, social support might be a positive factor for CD patients in distress [38]. In China, 30.6% of patients spend half of their income on treatment. This statistic suggests that more social investment should be applied to CD in our country. Social investment seems to generate some financial burden for the society, but it is cost-effective compared to the burden for every patient [34].

Our findings have two implications. First, this was the first study using the EQ-5D-5L to evaluate HRQoL in people with CD in China. Our results thus could be compared with those derived from other HRQoL instruments. Second, the impaired HRQoL and financial stress detected suggest that CD greatly disturbs patients and their families. Our study offers useful information to policy-makers for developing better strategies for CD patients.

There were also some limitations. First, the population lacked a control group, and the study was unable to compare health outcomes between patients and healthy subjects, which could make the study unconvincing. In addition, the sample size was modest, and the number of people in some subgroups varied greatly, weakening the generalizability of the results. Last, the cross-sectional design and single-centre nature of this study may have led to certain biases.

## Conclusion

In our study, CD patients reported worse health in the pain/discomfort and anxiety/depression dimensions of the EQ-5D-5L. Being in an active phase of disease and using antibiotics had detrimental impacts on HRQoL and health utility. Future research should overcome the limitations of our current study to provide useful information for clinical interventions in CD patients.

## Data Availability

The dataset used for this research will be available from the corresponding author upon reasonable request.

## Abbreviations

CD: Crohn’s Disease
HRQoL: Health-related Quality of Life
EQ-5D-5L: EuroQol 5-dimensions Instrument with 5-level Scale
BMI: Body Mass Index
SD: Standard Deviation
DALY: Disability-adjusted Life-year
QALY: Quality-Adjusted Life Year
CTE: Computed Tomography enterography
MRE: Magnetic Resonance Enterography
CDAI: Crohn’s Disease Active Index
ESR: Erythrocyte sedimentation rate
CRP: C-reactive protein
AIC: Akaike information criterion
VIF: Variance inflation factor
